# An immune, stroma, and epithelial–mesenchymal transition‐related signature for predicting recurrence and chemotherapy benefit in stage II–III colorectal cancer

**DOI:** 10.1002/cam4.5534

**Published:** 2023-01-11

**Authors:** Du Cai, Wei Wang, Min‐Er Zhong, Dejun Fan, Xuanhui Liu, Cheng‐Hang Li, Ze‐Ping Huang, Qiqi Zhu, Min‐Yi Lv, Chuling Hu, Xin Duan, Xiao‐Jian Wu, Feng Gao

**Affiliations:** ^1^ Department of Colorectal Surgery, The Sixth Affiliated Hospital Sun Yat‐sen University Guangzhou China; ^2^ Guangdong Institute of Gastroenterology Guangzhou China; ^3^ Guangdong Provincial Key Laboratory of Colorectal and Pelvic Floor Diseases, The Sixth Affiliated Hospital Sun Yat‐sen University Guangzhou China; ^4^ Department of Clinical Laboratory, Haining People's Hospital Jiaxing China; ^5^ Department of Gastrointestinal Endoscopy, The Sixth Affiliated Hospital Sun Yat‐sen University Guangzhou China; ^6^ Department of Colorectal Surgery Ningbo Medical Center Lihuili Hospital Ningbo China

**Keywords:** colorectal cancer, epithelial–mesenchymal transition, immune, prognosis, stroma

## Abstract

**Background:**

Debates exist on the treatment decision of the stage II/III colorectal cancer (CRC) due to the insufficiency of the current TNM stage‐based risk stratification system. Epithelial–mesenchymal transition (EMT) and tumor microenvironment (TME) have both been linked to CRC progression in recent studies. We propose to improve the prognosis prediction of CRC by integrating TME and EMT.

**Methods:**

In total, 2382 CRC patients from seven datasets and one in‐house cohort were collected, and 1640 stage II/III CRC patients with complete survival information and gene expression profiles were retained and divided into a training cohort and three independent validation cohorts. Integrated analysis of 398 immune, stroma, and epithelial‐mesenchymal transition (ISE)‐related genes identified an ISE signature independently associated with the recurrence of CRC. The underlying biological mechanism of the ISE signature and its influence on adjuvant chemotherapy was further explored.

**Results:**

We constructed a 26‐gene signature which was significantly associated with poor outcome in Training cohort (*p <* 0.001, HR [95%CI] = 4.42 [3.25–6.01]) and three independent validation cohorts (Validation cohort‐1: *p* < 0.01, HR [95%CI] = 1.70 [1.15–2.51]; Validation cohort‐2: *p* < 0.001, HR [95% CI] = 2.30 [1.67–3.16]; Validation cohort‐3: *p* < 0.01, HR [95% CI] = 2.42 [1.25–4.70]). After adjusting for known clinicopathological factors, multivariate cox analysis confirmed the ISE signature's independent prognostic value. Subgroup analysis found that stage III patients with low ISE score might benefit from adjuvant chemotherapy (*p* < 0.001, HR [95%CI] = 0.15 [0.04–0.55]). Hypergeometric test and enrichment analysis revealed that low‐risk group was enriched in thr immune pathway while high‐risk group was associated with the EMT pathway and CMS4 subtype.

**Conclusion:**

We proposed an ISE signature for robustly predicting the recurrence of stage II/III CRC and help treatment decision by identifying patients who will not benefit from current standard treatment.

## 1 INTRODUCTION

Recent data show that the mortality and incidence of colorectal cancer (CRC) rank second and third, respectively, which cause a significant burden on public health and economic development.^1^ In the past decades, fluorouracil‐based adjuvant chemotherapy has reduced the relapse rate and promoted the prognosis of patients with advanced CRC.^2^ Although current clinical guidelines recommend high‐risk stage II and stage III patients receive postoperative adjuvant chemotherapy, whether these patients can benefit from adjuvant chemotherapy remains controversial.^3^, ^4^, ^5^ Moreover, there are still approximal 30% stage II and 60% stage III CRC patients suffering from recurrence after standard treatment, which reflects the insufficiency of the current TNM stage‐based risk stratification system.^6^ Recent progress in molecular subtypes improved that molecular heterogeneity characterized by gene expression data can predict prognosis and guide treatment decisions.^7^, ^8^ However, the current classifier for the CMS subtype contained hundreds of genes, which makes it difficult to apply in routine clinical practice. Precision treatment for patients with stage II/III CRC relies on more effective and easily applied molecular biomarkers.

Research on tumor microenvironment (TME) has recently made significant strides, allowing us to gain a better understanding of how CRCs generate and develop. The components of the TME in CRC are quite complex, including tumor cells, immune cells, stromal cells, and other extracellular components.^9^ Several studies have found associations between TME and the prognosis of CRC. For example, immunoscore was found to be a candidate prognostic marker in CRC.^10^, ^11^ In some typical molecular subtyping studies, the high stroma infiltration subtype had a significantly worse outcome than other subtypes.^7^, ^12^ However, considering the heterogeneity of CRC and the complexity of the TME, the prognosis information provided by a single component is limited.

Besides the TME, epithelial–mesenchymal transition (EMT) also plays an essential role in tumor invasion and metastasis. The process of EMT endows the tumor cells with a more aggressive phenotype, which may result in a poor prognosis.^13^, ^14^, ^15^ Recent research has established an intrinsic link between EMT and TME. Some cytokines and chemokines secreted by TME cells are crucial for inducing EMT to promote tumor invasion and metastasis.^16^ However, whether the combination of TME and EMT‐related signals can bring better prognostic value remained unknown.

In this study, by integrating multi‐component of the TME and EMT‐related signals, we developed and validated an immune, stroma, and EMT (ISE)‐related gene signature for prognosis prediction in stage II/III CRC. The clinical utility of ISE signature was further validated in the in‐house COCC cohort (Clinical Omics Study of Colorectal Cancer in China). We believed the ISE gene signature can provide a robust risk‐stratification system beyond TNM‐stage and provide evidence for guiding treatment decisions in clinical practice.

## 2 MATERIALS AND METHODS

### 2.1 Public data source

This study included multiple gene expression datasets from the Cancer Genome Atlas (TCGA) and Gene Expression Omnibus (GEO) database for model training and validation. We use the largest GEO dataset GSE39582 as the training cohort. TCGA CRC was used as the validation‐1 cohort, and five GEO datasets with relatively small sample size (GSE14333, GSE37892, GSE17536, GSE39084, GSE33113) were merged as the validation‐2 cohorts. The “GEOquery” package was used to download all of the GEO gene expression profiles as well as the corresponding clinical data.^17^ TCGA RNA‐seq data with log2 transformed transcripts per million (TPM) were obtained from the Broad GDAC Firehose (http://gdac.broadinstitute.org/). Combat algorithm from the “sva” package^18^ and z‐score normalization was used to remove the batch effect.

### 2.2 In‐house clinical cohort

We used the COCC cohort, the CRC subproject of the ICGC‐ARGO (https://www.icgc‐argo.org/) as the independent validation‐3 cohort. All of the cases enrolled in this project were from the Sixth Affiliated Hospital of Sun Yat‐sen University. In this study, 208 stage II/III patients with complete RNA‐seq data and follow‐up data were incorporated. Total RNA was extracted by TRAzol from fresh frozen tissues. rRNA was removed by MGIEasy rRNA Depletion Kit (32 RXN, 1000005953) according to the instructions of manufacturer. The input for RNA‐seq library preparation was 500 ng per sample. Sequencing library was then generated based on conventional random primers method. Whole transcriptome sequencing was performed on the DNBSEQ‐T1 platform (BGI). HISAT was used to align the reads to the GRCh38/hg38 reference genome.^19^ Quantification of transcript expression was performed by RSEM.^20^

### 2.3 Identification of the ISE signature

Immune‐, stroma‐, and EMT‐related genes were identified from the published literature and MSigDB (The Molecular Signatures Database) (Table S1).^21^, ^22^, ^23^ To ensure that the gene signature can be detected stably, we only retained the top 50% expressed genes based on mean expression level. The LASSO algorithm was subsequently used to select the ISE genes included in the final model. The penalty parameter was selected at the minimum partial likelihood deviance, which was estimated by 10‐fold cross‐validation. The risk score was calculated with the following formula: ISE risk score = CD68 × 0.046 + FUCA1 × 0.055 + GZMB × (−0.101) + IRF8 × (−0.051) + KDM6B × 0.050 + LGALS9 × (−0.097) + MMP9 × (−0.170) + MX1 × (−0.053) + MICAL2 × 0.009 + STEAP1 × (−0.087) + PPARG × (−0.016) + AKR1C3 × 0.019 + LIPG × (−0.122) + COL4A1 × 0.013 + CXCL1 × (−0.007) + GJA1 × 0.058 + LAMC2 × 0.070 + MATN2 × (−0.050) + NID2 × 0.012 + NT5E × 0.059 + SAT1 × 0.089 + SDC4 × (−0.028) + SERPINH1 × 0.070 + TNFRSF12A × 0.016 + TPM4 × 0.018 + VEGFA × 0.077. To categorize patients into high‐ and low‐risk groups, the threshold of the risk score was determined using the time‐dependent receiver operating characteristic (ROC) curve.

### 2.4 Validating the prognostic value of ISE signature

Survival analysis was performed to assess the ISE signature's prognostic value in stage II/III CRC in the training cohort and three independent validation cohorts. Age, sex, tumor location, TNM stage, mismatch repair (MMR) status, and ISE signature were included in the univariate analysis. The subsequent multivariate analysis tested the independent prognostic significance of ISE signature on variables with significant *p* < 0.05 in the univariate analysis. Subgroup analysis was carried out to explore the link between ISE signature and benefit from adjuvant chemotherapy. Furthermore, we also compared the performance of ISE signature with clinicopathological factors and previously published multi‐gene signature in predicting the recurrence of CRC.

### 2.5 Functional analysis

We used gene set enrichment analysis (GSEA) based on differentially expressed genes in the high‐risk and low‐risk groups to derive an underlying biological interpretation of the ISE signature. We further investigate the association between ISE signature and CMS subtypes by hypergeometric test.

### 2.6 Statistical analysis

The log‐rank test and Kaplan–Meier curves were utilized to examine the relationship between the ISE signature and 5‐year disease‐free survival. Univariate and multivariate regression visualized by forest plot were conducted to select the prognostic factors. *C*‐index and ROC curve were used to compare the performance of the ISE signature with clinicopathological factors and published gene panel. The meta‐estimate C‐index was calculated by survcomp package.^24^ GSEA was carried out by the R package called “HTSanalyzeR”.^25^ Kruskal–Wallis test and hypergeometric test were used to evaluate the association between the ISE signature and CMS subtypes. *p* < 0.05 was deemed statistically significant. R language was used to conduct all statistical analyses (Version 3.6.1).

## 3 RESULTS

### 3.1 Identification of a 26‐geneISE signature for prognosis prediction of stage II/III CRC

**FIGURE 1:**
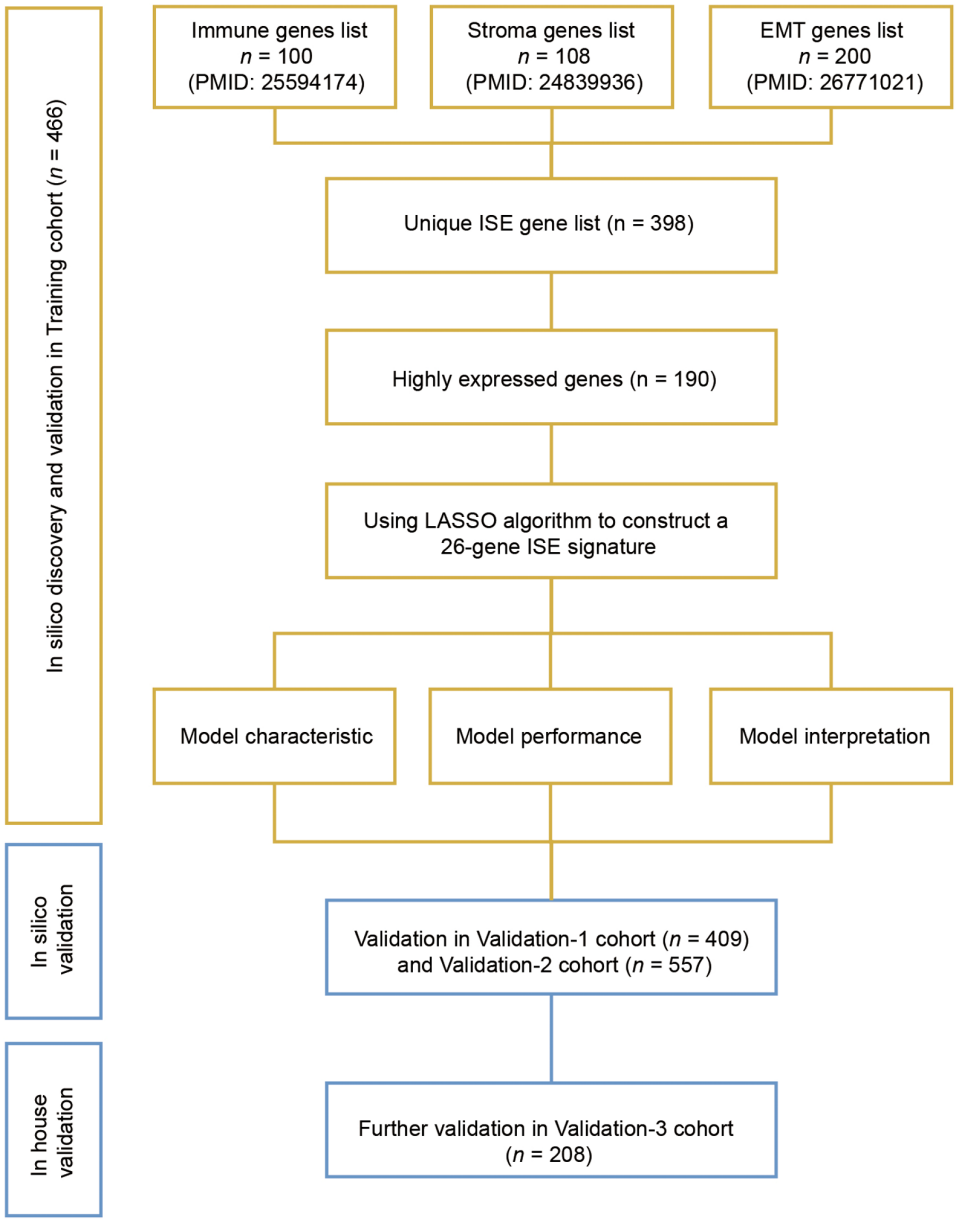
Overall workflow of this study.

**FIGURE 2:**
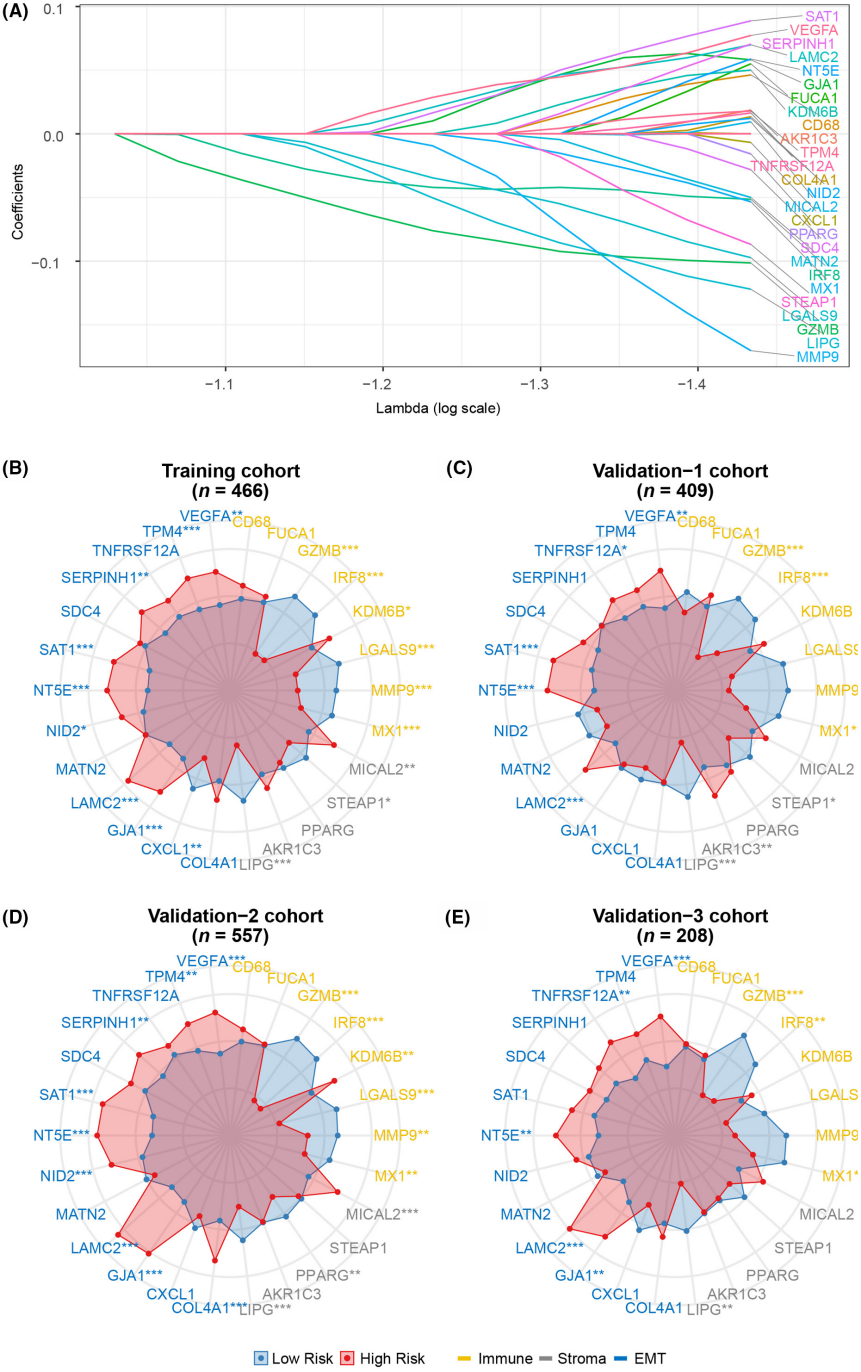
The construction and characteristic of the ISE signature. (A) The 26‐gene ISE signature was identified by the LASSO algorithm. (B–E) The scaled expression level of immune (yellow), stroma (gray), and EMT (blue) related genes between high‐ and low‐risk group. Wilcoxon test was used to determine the statistical difference of ISE genes between the two risk groups. *p < 0.05, **p < 0.01, ***p < 0.001.

Seven public datasets and one in‐house datasets including 1,640 stage II/III CRC patients were enrolled and categorized into a training cohort (*n* = 466) and three independent validation cohorts (*n* = 409, 557 and 208, respectively). The clinical characteristics of all patients were showed in Table S2. According to our study design (Figure 1), 190 genes with expression level higher than the median value were selected from 398 immune‐, stroma‐, and EMT‐related genes, and a 26‐gene ISE signature was identified by LASSO cox regression (Figure 2A). The risk scores were calculated by weighted summation of these 26 genes (Figure S1). All patients were categorized into low‐ and high‐risk groups based on the optimal threshold determined in the training cohort (Figure S2, Table S3). Among these 26 genes, most of the immune genes overexpressed in the low‐risk group, while the EMT‐related genes tend to express higher in the high‐risk group (Figure 2B–E).

### 3.2 ISE signature was significantly associated with the prognosis of stage II/III CRC

**FIGURE 3:**
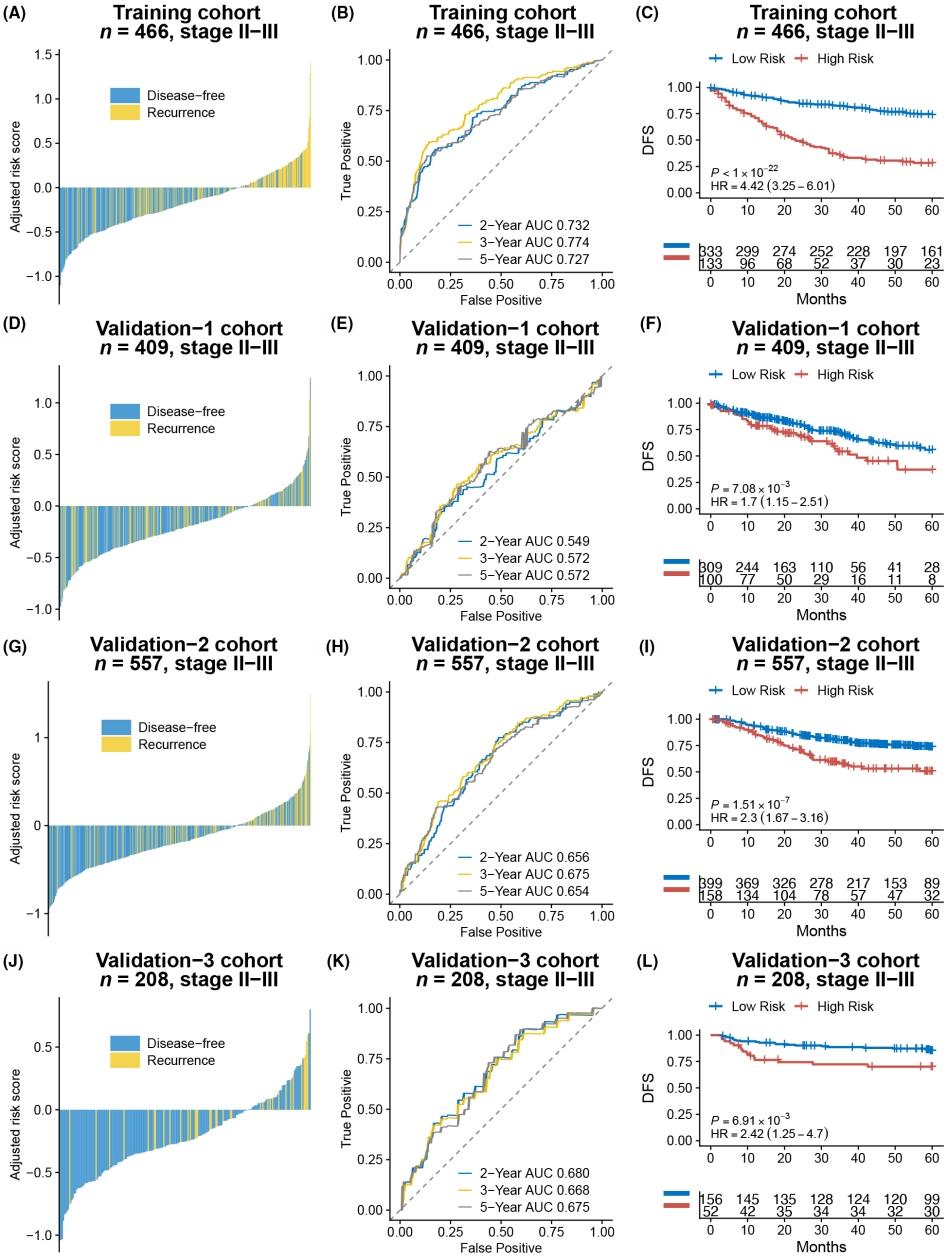
The association between ISE signature and the outcome of stage II/III colorectal cancer patients. The waterfall plots showed the relationship between ISE risk score and recurrence status (A, D, G, J). Time‐dependent ROC curves showed the discriminative ability of ISE signature for 2‐, 3‐, and 5‐ year recurrence status (B, E, H, K). The Kaplan–Meier curves revealed a significant association between the ISE signature and disease‐free survival (DFS) in four cohorts(C, F, I, L).

**FIGURE 4:**
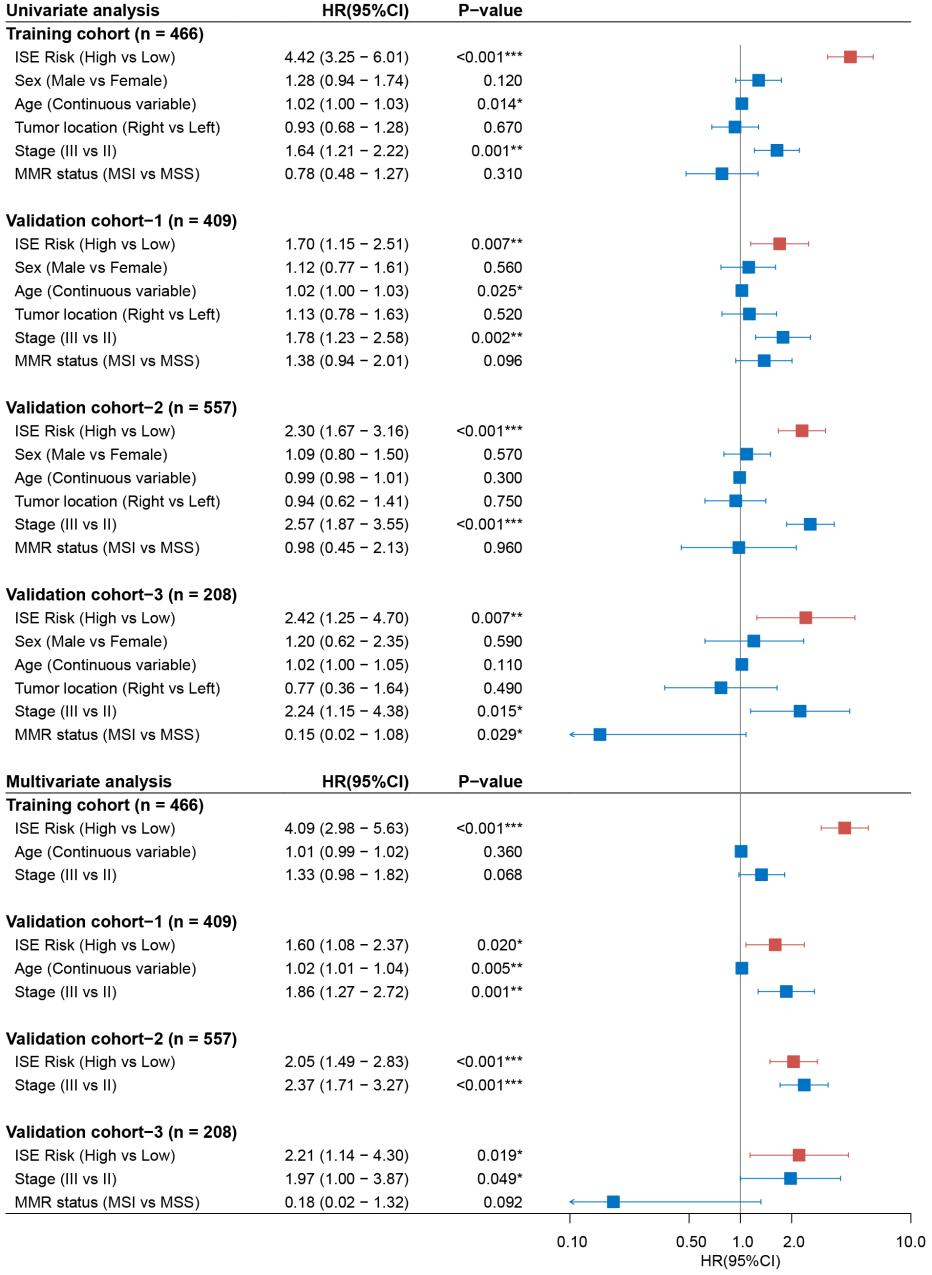
Univariate and multivariate analysis of ISE signature and clinicopathological factors for disease‐free survival (DFS) of stage II/III colorectal cancer. Age was used as continuous variable. *p < 0.05, **p < 0.01, ***p < 0.001.

The Kaplan–Meier curves showed that patients in high‐risk group had significantly worse outcome than low‐risk group (Training: *p* < 0.001, HR [95% CI] = 4.42 [3.25–6.01]; Validation cohort‐1: *p* < 0.01, HR [95% CI] = 1.70 [1.15–2.51]; Validation cohort‐2: *p* < 0.001, HR [95% CI] = 2.30 [1.67–3.16]; Validation cohort‐3: *p* < 0.01, HR [95% CI] = 2.42 [1.25–4.70]) (Figure 3). Multivariate analysis showed that ISE signature remained significant in all cohorts after adjusting for other clinicopathological factors (Training cohort: *p* < 0.001, HR [95% CI] = 4.09 [2.98–5.63]; Validation‐1 cohort: *p* = 0.02, HR [95% CI] = 1.60 [1.08–2.37]; Validation‐2 cohort: *p* < 0.001, HR [95% CI] = 2.05 [1.49–2.83]; Validation‐3 cohort: *p* = 0.019, HR [95%CI] = 2.21 [1.14–4.30]) (Figure 4).

### 3.3 ISE signature could be used to predict the benefits of adjuvant chemotherapy

**FIGURE 5:**
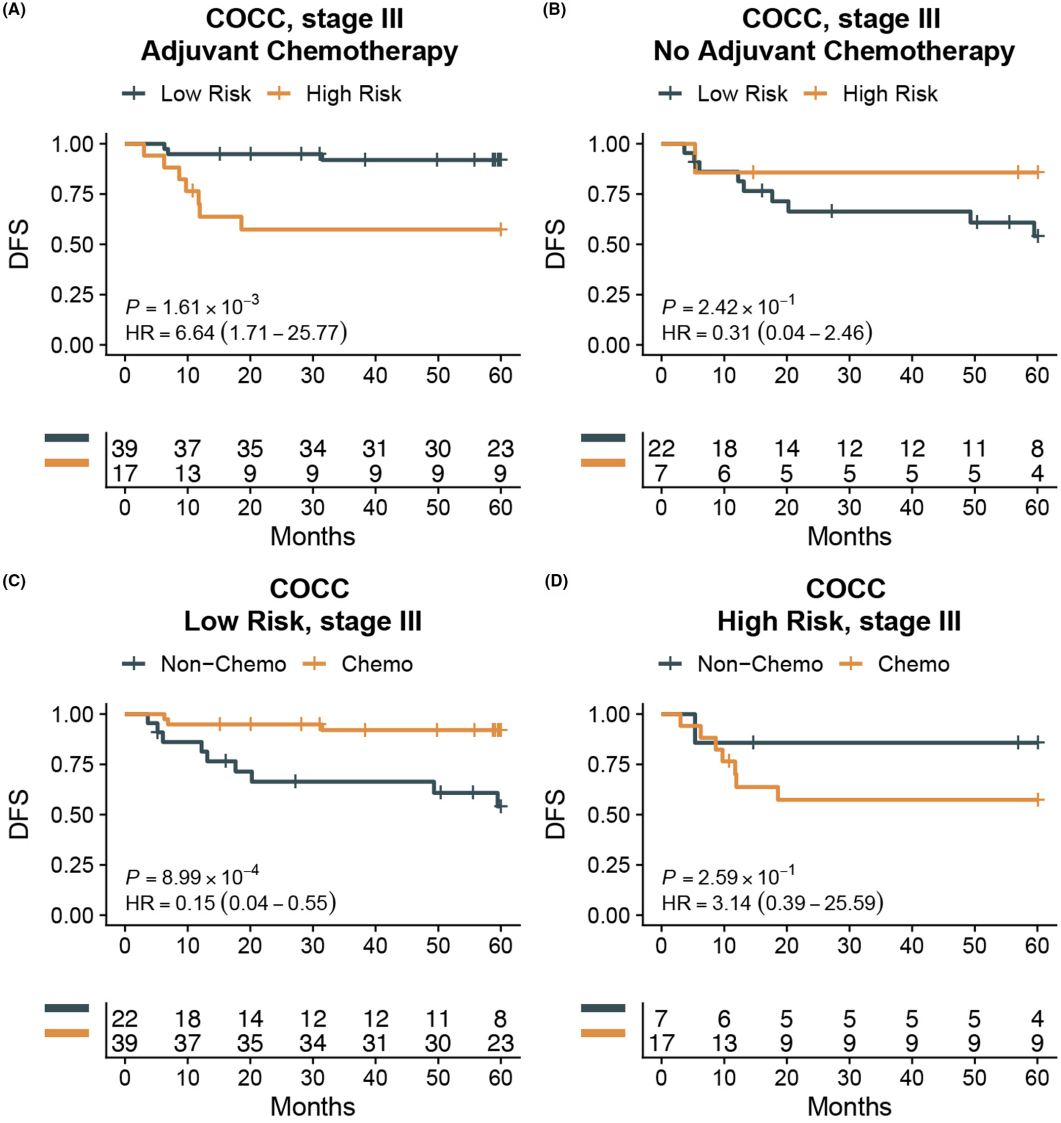
Subgroup analysis in COCC cohort showed the association between ISE signature and benefit from adjuvant chemotherapy in stage III colorectal cancer. (A) For patients who had received adjuvant chemotherapy, high‐risk group had significant worse outcome. (B) No significant different outcome was observed in low‐ and high‐risk group for those without adjuvant chemotherapy. (C) Patients in low‐risk group could significantly benefit from adjuvant chemotherapy. (D) No significant benefit was found in high‐risk group from adjuvant chemotherapy.

To further investigate the value of ISE signature in adjuvant chemotherapy, our in‐house COCC cohort was used for subsequent subgroup analysis. The association of ISE score with other clinicopathologic features was shown in Table S5. Subgroup analysis found that low‐risk patients still had a significant better outcome than high‐risk patients in stage III patients who had received adjuvant chemotherapy (*p* = 0.002, HR [95% CI] = 6.64 [1.71–25.77]), whereas no obvious difference was observed in subgroup patients without adjuvant chemotherapy (*p* = 0.242, HR [95% CI] = 0.31 [0.04–2.46]) (Figure 5A,B). Besides, for low ISE‐risk stage III patients, patients who underwent adjuvant chemotherapy had a better prognosis than those who did not have adjuvant chemotherapy (*p* < 0.001, HR [95% CI] = 0.15 [0.04–0.55]) (Figure 5C). However, for stage II and high ISE‐risk stage III patients, no significant different outcome was found between patients did or did not undergo adjuvant chemotherapy (Figure 5D, Figure S3). These results suggested that adjuvant chemotherapy may be beneficial for patients with low‐risk stage III CRC.

### 3.4 ISE signature performed better than Oncotype DX and could be a supplement to TNM stage

**FIGURE 6:**
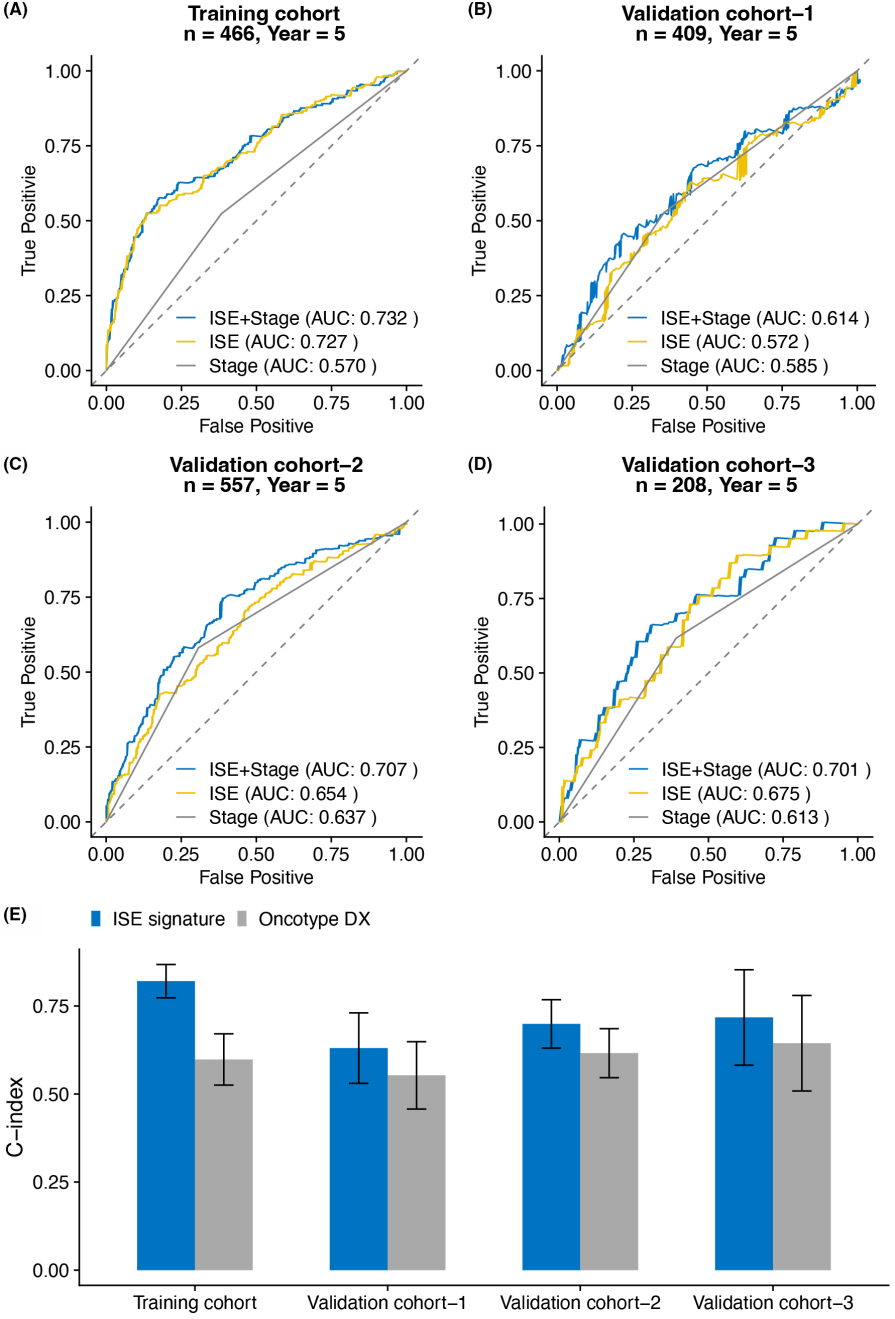
The performance of ISE signature comparing to clinicopathological factor and published gene expression‐based panel. (A–D) Time‐dependent ROC curves showed that the combination of ISE signature and TNM stage achieved higher AUC than using TNM stage alone. (E) C‐index showed that the ISE signature had a better prognostic value than Oncotype DX. ISE: immune, stroma, and epithelial‐mesenchymal transition. ns: not significant.

According to the results of multivariate analysis and prior knowledge, TNM stage was an important prognostic factor in CRC. To further explore the clinical value of ISE signature, we compared the ISE signature with TNM stage and Oncotype DX panel.^26^ While the ISE signature achieved similar discriminative value of TNM stage, combination of ISE signature and TNM stage (AUC = 0.732, 0.614, 0.707, and 0.701, respectively) performed better than using TNM stage alone (AUC = 0.570, 0.585, 0.637, and 0.613, respectively) (Figure 6A–D). When comparing to the well‐known multi‐gene panel Oncotype DX (meta‐estimated *C*‐index: 0.602 [0.519–0.684]), the ISE signature still achieved higher *C*‐index (meta‐estimated *C*‐index: 0.744 [0.656–0.832], *p* < 0.01) (Figure 6E; Table S4).

### 3.5 Low‐risk group was enriched in immune pathway and high‐risk group was associated with the EMT pathway and CMS4 subtype

**FIGURE 7:**
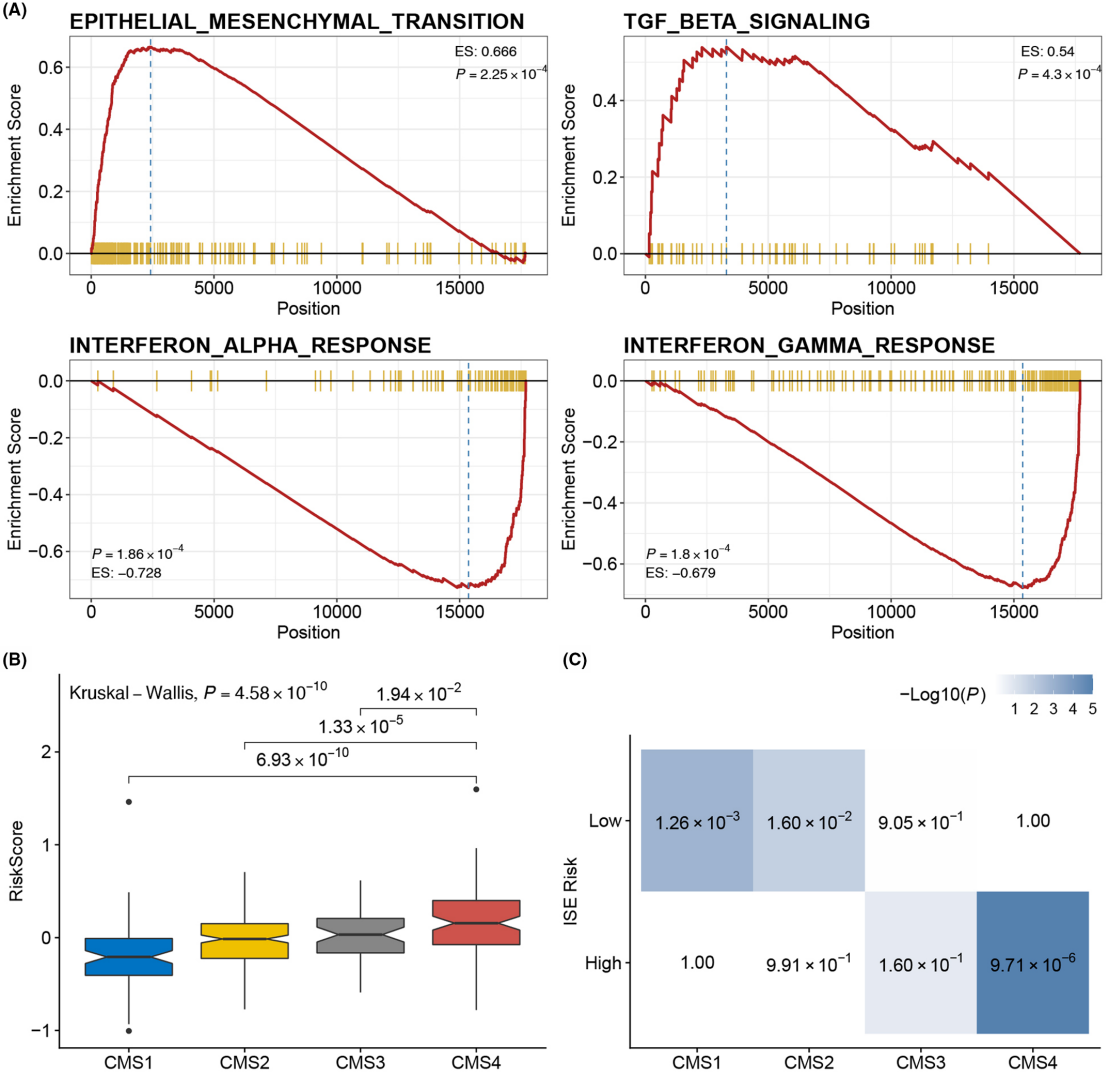
The biological mechanism of ISE signature and association with CMS subtypes. (A) Enrichment analysis showed that TGF beta signaling and epithelial‐mesenchymal transition (EMT) pathways were upregulated in the high‐risk group, while immune‐related pathways like interferon‐alpha response and interferon‐gamma response pathway were downregulated in the high‐risk group. (B) Boxplot showed that the risk score was significantly higher in CMS4 subtype. (C) The hypergeometric test revealed a significant association between the high‐risk group and the CMS4 subtype, whereas the low‐risk group was linked to the CMS1 and CMS2 subtypes.

GSEA analysis showed that some pathway associated with tumor invasion and metastasis such as EMT and TGF beta signaling pathway were upregulated in the high‐risk group. However, some immune‐related pathways like interferon‐gamma response and interferon‐alpha response were downregulated in the high‐risk patients (Figure 7A). Considering the stroma component of the ISE signature, we further investigate the relationship between the ISE signature and CMS4 subtype, which was characterized as high stroma infiltration. The results showed that the risk score was significantly higher in CMS4 subtypes (Figure 7B). Besides, hypergeometric test found that the high‐risk group was overrepresented in the CMS4 subtype (*p* < 0.001, Figure 7C).

## 4 DISCUSSION

Adjuvant chemotherapy was recommended as routine treatment for high‐risk stage II and all stage III CRC patients. However, only 20% of stage III patients can benefit from postoperative chemotherapy, resulting in the rest 80% of patients suffering from unnecessary drug toxicity.^27^ Besides, how to choose the appropriate stage II patients for chemotherapy is still controversial.^28^ As a result, a better risk stratification system for precision treatment of stage II/III CRC is an absolute necessity. Herein, we propose an integration of tumor environment and EMT‐related signals, both of which were associated with tumor invasion and metastasis, to improve the prediction of recurrence for CRC. A 26‐gene ISE signature was constructed which can classify stage II/III CRC individuals into different risk groups. Four cohorts with large sample size, including seven public datasets and one in‐house cohort, were used to test the predictive value of the ISE signature. The results of univariate and multivariate analyses confirmed that the ISE signature could be an independent prognostic factor for CRC patients in stage II/III. Comparing to Oncotype DX, the ISE signature showed superior prognostic value, and the ISE signature in combination with TNM stage outperformed the TNM stage alone. Subgroup analysis found that only low ISE risk stage III patients could really benefit from adjuvant chemotherapy, suggested that current indications of chemotherapy based on the TNM stage and clinicopathological factors were insufficient.

EMT is a well‐known pathway associated with the invasive and metastatic phenotype in CRC.^29^ Additionally, the importance of TME in CRC has also been increasingly recognized.^30^ Some studies have tried to quantify immune infiltration status for predicting the prognosis of CRC, such as Immunoscore and immune‐related gene signature.^11^, ^31^, ^32^ Interestingly, most of the immune genes were overexpressed in the low‐risk group, whereas EMT‐related genes show a reverse pattern. Moreover, GSEA analysis found that several tumor malignancy‐related pathways including EMT and TGF‐beta were upregulated, while immune‐related pathways were downregulated in the high‐risk group. In contrast to the favorable impact of immune infiltration, high stroma infiltration was considered to be related to poor outcome.^33^ Hypergeometric test showed a strong association between the high‐risk group and the CMS4 subtype, and the latter was also known as the subtype with high stroma infiltration and worst prognosis. Collectively, the above analyses provide a biological interpretation of the ISE signature that the high ISE risk group shows a pattern of low immune infiltration, high stroma infiltration, and upregulation of EMT pathway.

Considering that the formation and progression of tumors involve the dysregulation of multiple pathways, such a combination can provide extra prognostic information. Our results confirmed that the ISE signature was a robust biomarker for the recurrence prediction and indication of adjuvant chemotherapy of CRC which can provide evidence for individualized treatment in clinical practice. Concerning the limitation of our study, this study used the data generated from the retrospective samples for analyses. Therefore, prospective studies are needed to reduce bias in future studies. We are also interested in better integrating TME and EMT‐related prognostic information through multimodal data, such as the fusion of sequencing data and medical image data in the future study.

## 5 CONCLUSIONS

In conclusion, we propose a ISE signature in combination with immune, stroma, and EMT‐related genes. Our ISE signature can robustly stratify stage II/III patients into distinct risk groups and identify those who would benefit from adjuvant chemotherapy, which can facilitate clinicians to make an individualized treatment strategy.

## AUTHOR CONTRIBUTIONS

**Du Cai:** Formal analysis (equal); investigation (equal); visualization (equal); writing – original draft (equal). **Wei Wang:** Formal analysis (equal); investigation (equal); visualization (equal); writing – original draft (equal). **Min‐Er Zhong:** Data curation (equal); resources (equal); writing – original draft (supporting). **Dejun Fan:** Data curation (equal); resources (equal); writing – original draft (supporting). **Xuanhui Liu:** Data curation (equal); resources (equal); writing – original draft (supporting). **Cheng‐Hang Li:** Data curation (equal); formal analysis (equal); software (equal). **Ze‐Ping Huang:** Data curation (equal); formal analysis (equal); validation (equal). **Qiqi Zhu:** Data curation (equal); formal analysis (equal); validation (equal). **Min‐Yi Lv:** Data curation (equal); formal analysis (equal); validation (equal). **Chuling Hu:** Data curation (equal); formal analysis (equal); validation (equal). **Xin Duan:** Data curation (equal); formal analysis (equal); methodology (equal); software (equal). **Xiaojian Wu:** Conceptualization (equal); funding acquisition (equal); project administration (equal); supervision (equal); writing – review and editing (equal). **Feng Gao:** Conceptualization (equal); funding acquisition (equal); project administration (equal); supervision (equal); writing – review and editing (equal).

## FUNDING INFORMATION

This study was supported by Guangzhou Basic and Applied Basic Research Fund (No. 202102020820, FG), the National Natural Science Foundation of China (No. 82002221, FG), the Sun Yat‐sen University 100 Top Talent Scholars Program – China (No. P20190217202203617, FG), The Sixth Affiliated Hospital of Sun Yat sen University Start‐up Fund for Returnees (no. R20210217202501975, FG), Project funded by China Postdoctoral Science Foundation (no. 2020M683121, MZ), National Natural Science Foundation of China (no. 81972212, XW), Natural Science Foundation of Guangdong Province, China (no. 2019A1515010063, XW).

## CONFLICT OF INTEREST

All authors declare no competing interest.

## ETHICAL APPROVAL

This study complies with the Declaration of Helsinki and was approved by the Medical Ethics Committee of the Sixth Affiliated Hospital of Sun Yat‐sen University.

## CONSENT FOR PUBLICATION

This study has obtained waiver of written informed consent granted by the Medical Ethics Committee.

## THE LIST OF COCC WORKING GROUP

Xin‐Juan Fan, Department of Pathology, The Sixth Affiliated Hospital of Sun Yat‐sen University, Guangzhou, China; Jia Ke, Department of Colorectal Surgery, The Sixth Affiliated Hospital of Sun Yat‐sen University, Guangzhou, China; Kui Wu, Lan‐Xin Zhou, Shu‐Zhen Luo, Dong‐Bing Liu, BGI‐Shenzhen, Shenzhen, China; Xin Wang, Tan Wu, Zhong‐Xu Zhu, Department of Biomedical Sciences, City University of Hong Kong, Hong Kong, China; Rong‐Hui He, Yidu Cloud Technology Co., Ltd, Beijing, China.

## Supporting information


Figure S1.

Table S1.

Table S2.

Table S3.

Table S4.

Table S5.

## Data Availability

The public datasets used in this study are available from Gene Expression Omnicbus (https://www.ncbi.nlm.nih.gov/geo/) and Broad GDAC Firehose (http://gdac.broadinstitute.org/). The other data supporting the findings of this study are available upon reasonable request from the corresponding authors (FG).
