# Supplementary material for: An immune, stroma, and epithelial–mesenchymal transition‐related signature for predicting recurrence and chemotherapy benefit in stage II–III colorectal cancer
Source: Cancer Med. 2023 Jan 11;12(7):8924–36. doi: 10.1002/cam4.5534 (PMC10134284; doi:10.1002/cam4.5534)
Supplement: Supplementary file 1 — Figure S1. [file CAM4-12-8924-s006.docx]

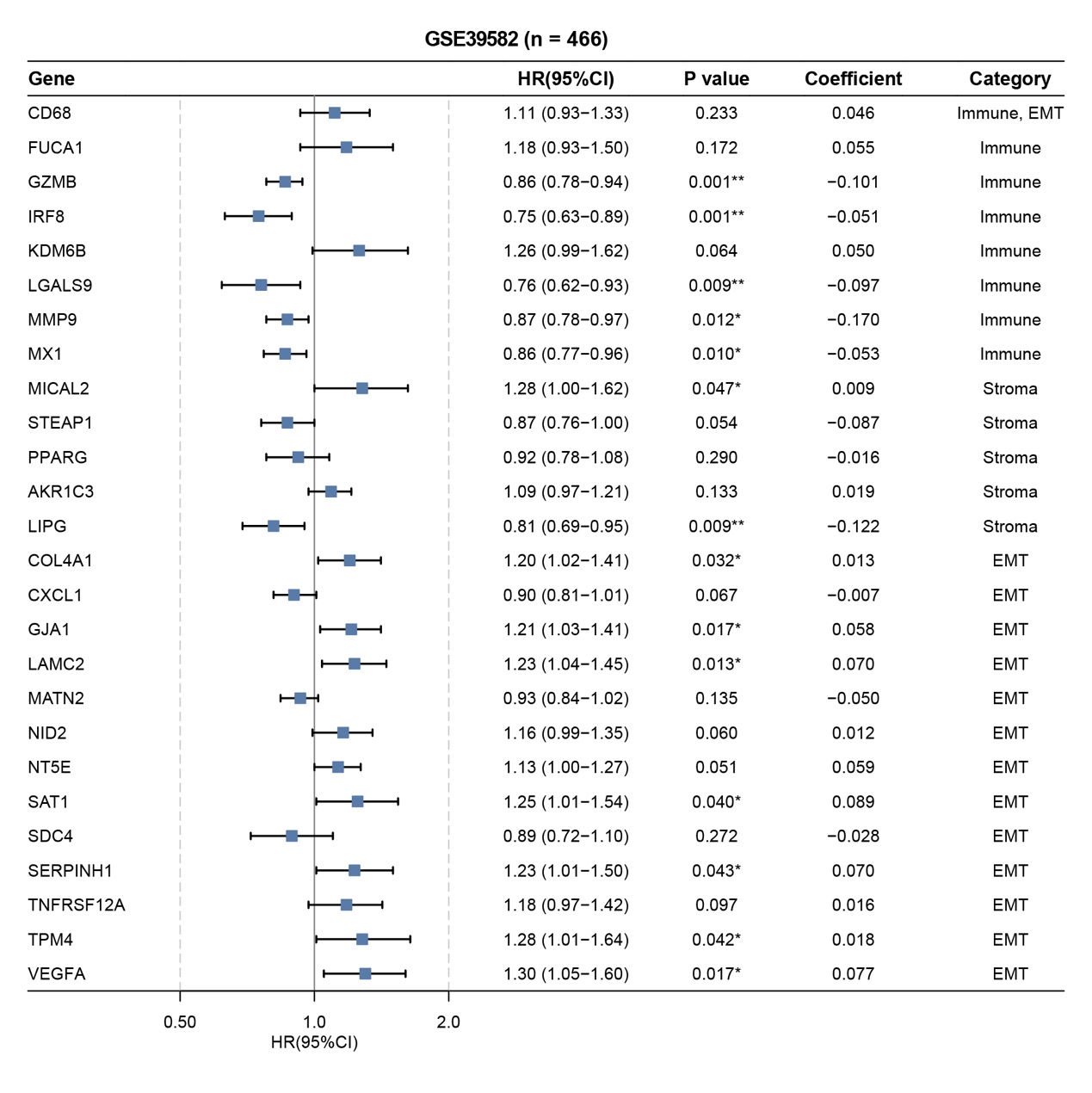


**Supplementary Fig. S1.** The list of 26 immune, stroma and epithelial mesenchymal transition related genes in ISE gene signature.

**Supplementary Fig. S2.** The optimal cutoff point was determined by the time dependent ROC curve in the training cohort.


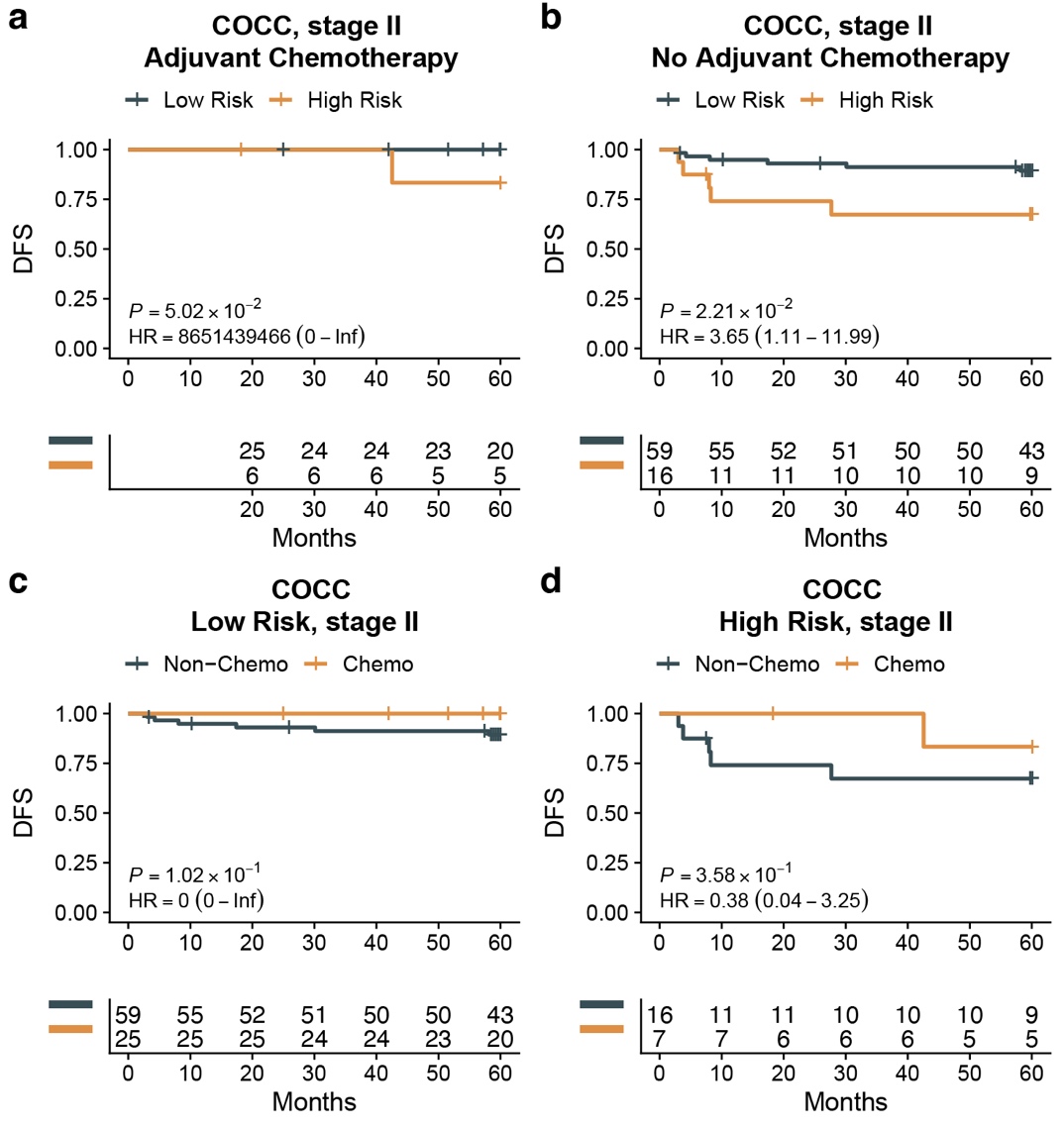


**Supplementary Fig. S3.** The association of ISE signature and adjuvant chemotherapy om stage II colorectal cancer patients.
